# Design of Mathematical Model and Selected Coefficient Specifications for Composite Materials Reinforced with Fabric from Waste Tyres

**DOI:** 10.3390/ma16145046

**Published:** 2023-07-17

**Authors:** Stella Hrehova, Lucia Knapčíková

**Affiliations:** Department of Industrial Engineering and Informatics, Faculty of Manufacturing Technologies with a Seat in Prešov, Technical University of Košice, Bayerova 1, 080 01 Prešov, Slovakia; lucia.knapcikova@tuke.sk

**Keywords:** PVB, composite materials, absorption, Matlab, mathematical model

## Abstract

Polyvinyl butyral (PVB) is a thermoplastic resin commonly used as an interlayer material in laminated glass. Combined with textile fibres from worn tyres, PVB can produce a composite material with unique properties. One such property is absorption. Absorption in textile fibre composite materials refers to the ability of the material to absorb or retain moisture or other fluids. The presence of moisture or liquids can affect the properties of composite materials, such as their strength, stiffness, and dimensional stability. As a result of the physical and chemical action of the environment, corrosion of plastics occurs and manifests itself in changes in appearance, surface quality, weight, dimensions, and also in changes in other properties. This paper investigates four composite materials with different fabrics content. The aim of this paper is to propose a generalized mathematical model for absorption values so that, with different ratios of textile fibre in the material, its quality indicators are at a sufficient level. Our study will proceed from the assumption that by changing the values of the individual coefficients of the selected model based on their analysis, we will achieve the required qualitative indicators.

## 1. Introduction

Composite materials are currently and increasingly used in various fields. Achieving maximum improvements in properties is achieved by a suitable choice of filler and matrix. The resulting properties of polymer composite materials are the result of the influence of several factors [1]. Among the most important are the following:

- Physical and chemical characteristics of the filler (chemical composition, structure, particle size and size distribution, and specific surface area);
- Physical and chemical characteristics of the matrix (chemical structure, molecular weight, and supramolecular structure);
- The shape of the filler;
- Amount of filler content in the composite;
- Method of preparation of composites;
- Character of the interphase interface [1].

Compared to metal and ceramic composites, fibre-reinforced polymer composites have many advantages [2]. The most important ones include relatively easy production and processing, resistance to corrosion, etc. [1,3] Plastics are not subject to electrochemical corrosion, such as metal corrosion, except for varying degrees of oxidative damage due to the action of oxygen, ozone, and an oxidizing medium in the environment, which is closely related to the macromolecular structure of plastics. Plastic molecules are composed of saturated chemical valence bonds, lack of free electrons, or moving ions forming an electrochemical effect with the medium, so electrochemical corrosion does not occur. In this sense, plastics are resistant to corrosion. These are most plastics against acids, bases, and other dielectrics with corrosion resistance. However, the special structure of plastic macromolecules is never destroyed, nor can any medium destroy it. In addition to oxidative damage in the atmosphere, in some cases below, due to the effects of acid, alkali, salt and other aqueous solutions (including oxidizing agents), and other chemicals, macromolecular materials, such as plastics, can be damaged from the surface quickly or slowly, which can actually be called the chemical aging of plastics. The basic undesirable phenomenon that occurs with polymers is a temporary change in surface electrical resistance caused by condensed water, which reduces the surface electrical resistance together with dust. Other factors are temperature changes and sunlight. Due to the influence of UV radiation, their degradation occurs, which manifests itself in the brittleness of the plastic, a change in colour, and other properties [3]. Plastics are not very resistant to short-wave radiation. Corrosion is manifested by a change in the colour of the material, and changes in macromolecules, which are manifested by the aging of the material. As a result of the increased temperature, plastics lose their flexibility and strength. Absorption values and intensity of light absorbed by a sample are generally measured as a function of wavelength. It will thus provide important information about the electronic structure of the atom or molecule of the particular material being analysed. Depending on the absorption values in the measurement sample, the results can also provide important insight into other material properties, such as sample concentration, phase changes, or changes in material composition [4]. The surface of the material becomes rough and acquires a greater ability to absorb moisture. The use of secondary raw materials in producing composite materials plays an important role in meeting EU goals, namely reducing energy consumption and producing new materials [3]. Existing and potential users are inclined to use secondary materials, compared to traditional ones, they often find that it is significantly more economical and saves the environment [3,5]. Effective recycling of waste materials, i.e., recycling products that try to copy the properties of natural materials. Polyvinyl butyral, used as a matrix in manufacturing composite materials, was created during the recycling of car glasses, where it is added as an admixture to the safety film between the individual layers of glasses. PVB film has several excellent properties, such as high tensile strength, impact resistance, transparency, and flexibility, which is particularly useful in producing safety glass [3]. Due to the content of alcohol, ester, and acetate bonds, PVB films can hold the glass firmly, even if the glass breaks. The glass adheres to the interlayers of the PVB film and thus prevents breakage [5]. It has been proven on the market that these materials are considered the most suitable and usable for a wide range of applications [6].

## 2. Materials, Methods, and Tools

### 2.1. Materials Characterization

**Figure 1:**
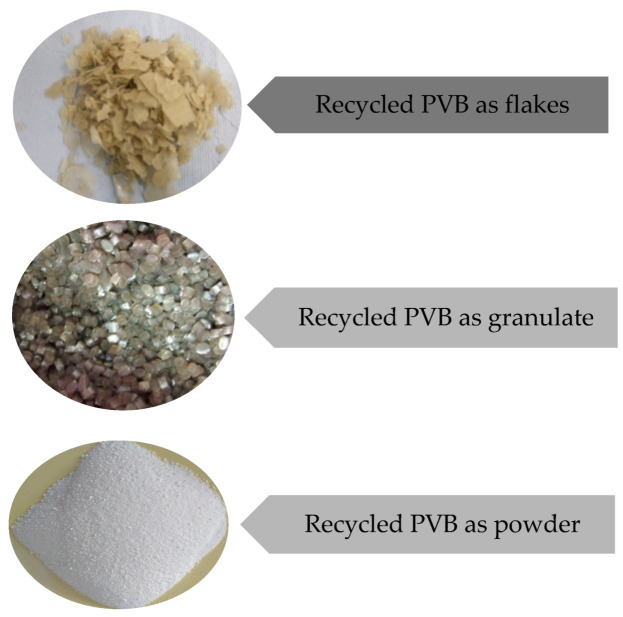
Recycled polyvinyl butyral and its form.

Polyvinyl butyral (PVB) is the most important polyvinyl acetal, as it accounts for around 90% of production. It is used as the main input material for the production of laminated automotive safety glass due to its excellent optical transparency, adhesive properties, toughness, and flexibility [7], but it is also used in other areas, especially in high-rise buildings. PVB is produced by several European and global companies [5]. The main applications of PVB films, PVB resins, are used for producing coatings, structural adhesives, dry toner paints, and as a binder for ceramics and composite fibres (Figure 1).

**Figure 2:**
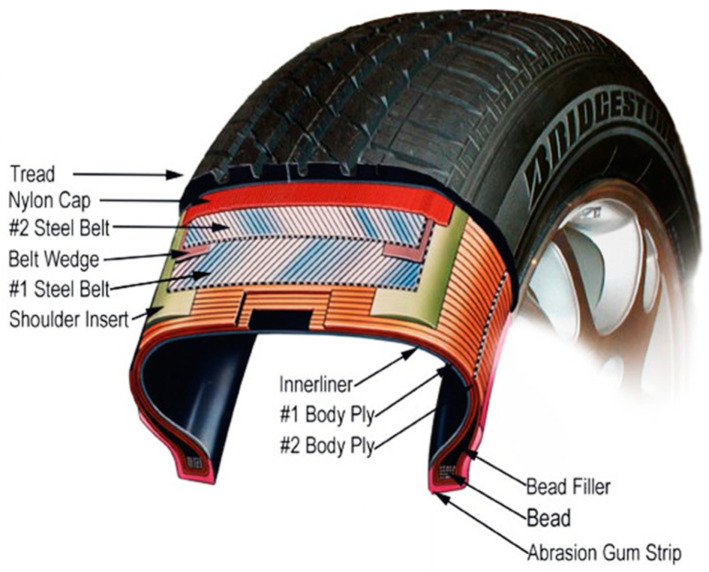
Basic tyre and its components [6].

The first basic method for making a composite is mixing composites. This method prepares mixtures of two or more polymer materials with non-polymeric additives (e.g., fillers, plasticizers, etc.) [8]. Generally, every vehicle needs a complete replacement of all four tyres every eight years. It depends mainly on the number of kilometres and the way of driving [3]. The priority of waste management with waste tyres is focused on waste tyres (Figure 2). Waste-processing facilities are constructed with a system of recycling waste components with regard to ecological solutions [8]. By supporting and applying the circular economy to production enterprises, new and more appropriate methods of processing waste tyres are developed using conventional and unconventional processing technologies. The mere use of waste tyres for recycling does not make sense [8].

**Figure 3:**
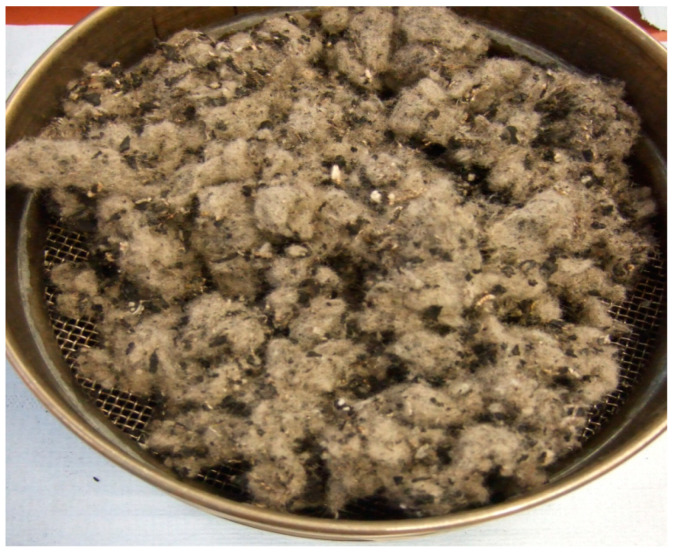
Vibrating screen cleaning process of fabrics.

Nowadays, Europe oversees tyre recycling for more than 100 independent companies across the continent. The companies collect and process data on the recovery or disposal of waste tyres [8]. One of the leading organizations is ETRA (European Tyre Recycling Association), which collects, processes, and evaluates data and informs residents about new possibilities for using secondary raw materials to produce a commodity [9]. From the current state, it is known that technologies are devoted to processing waste tyres by disintegrating and separating individual parts, which are mainly made rubber. Using the separation–sieving analysis, three important components were achieved [9]:

- Rubber;
- Steel;
- Fabric component (Figure 3).

The main function of fabrics in tyres is to provide stable vehicle performance through the required conditions [8,9]. The fabric composition mainly depended on cellulose, polyamide PA 6, and polyamide PA 6.6. Polyester terephthalate (abbreviated form PET), polyethene phthalate (PEN), and aramid (abbreviated form of aromatic polyamides) are listed as fibres in high-performance tyres [10].

#### Composites Manufacturing Process

For the preparation of the composite, continuous mixing of individual mixtures was used, which took place on twin-screw mixing devices [4]. Subsequently, the homogenized mixture was pressed in a Brabender W 350 laboratory press, where the basic technological parameters for pressing composite materials were the following:

- Pressing pressure;
- Pressing temperature;
- Curing time [10].

The pressing pressure affects the quality of the surface of the moulding, causing shrinkage. It depends on the type of material, the geometry of the product, the preheating temperature, and the pressing temperature. It is usually in the range of 10 to 60 MPa [9]. The pressing moulds are heated by electric resistance heating at the pressing temperature, which is actually the temperature of the pressing mould. Pressing temperatures depend on the type of plastic, wall thickness, product geometry, preheating temperature, and range from 130 to 190 °C. The uniformity of the mould temperature is important [11]. After moulding, the composite material’s test samples were subjected to absorption monitoring. This knowledge of the material’s ability to absorb or retain moisture or other liquids is important. It is widely known that the presence of moisture or liquids can affect the properties of composite materials, such as their strength, stiffness, and dimensional stability [11]. In the literature, we can find studies that investigate the nature of water uptake by composite materials because the water molecules absorbed by these materials play the role of a plasticizer and can disrupt the fibre/resin structure and thus cause deterioration of the fibre/matrix interface [12].

**Figure 4:**
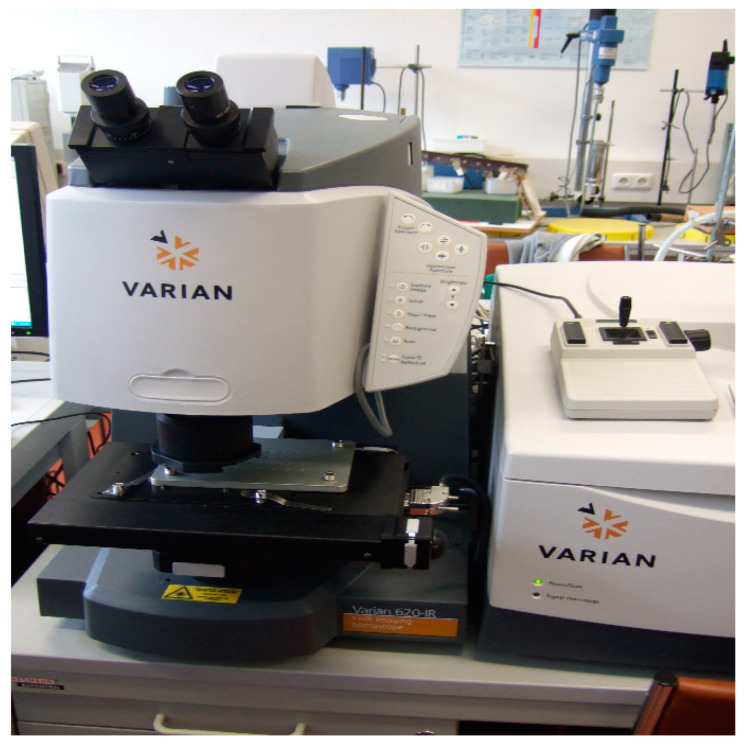
Absorption monitoring by VARIAN 620-IR.

Some types of liquid and gaseous substances have an aggressive effect on the surfaces of plastic products. This phenomenon is called corrosion. Corrosion of plastics manifests itself in changes in appearance, surface quality, weight, and dimensions and also in changes in other properties. Identifying the main components in the composite material, the characteristics of the structure and chemical composition of individual components and monitoring some reactions that take place on the surface of the tested material are very important. The samples were monitored on the VARIAN 620-IR device (Figure 4).

**Figure 5:**
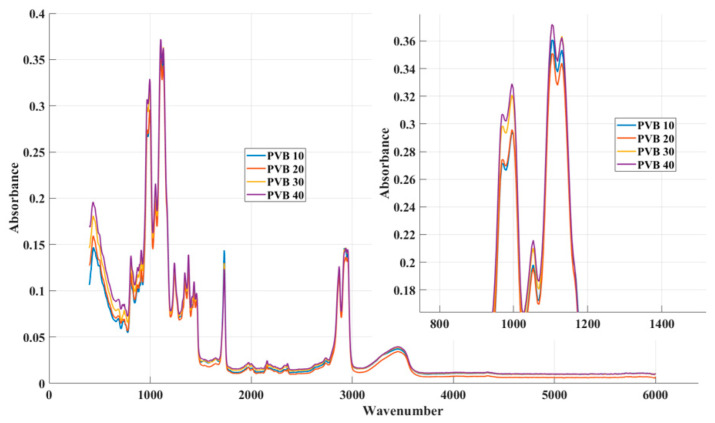
Measured absorption values with detail of the upper part.

The basic optical component of absorption monitoring is the interferometer, which consists of two mutually perpendicular mirrors and a beam splitter [9,10,11]. One of the mirrors has a fixed position, while the other moves along a specified path at a constant speed. The beam splitter is a semi-transparent plate made of a material that is transparent in the spectral region for which the beam splitter is intended [8]. IR radiation from the source falls on the beam splitter, which it is divided into two parts: One part falls on the fixed mirror and the other on the moving mirror. After reflection from the mirrors, the rays return to the beam splitter [11,13]. Since the path difference of both beams changes (depending on the position of the moving mirror), the beams interfere when returning to the beam splitter, and the signal arriving at the detector generates an interferogram. It contains information about the intensities for each wavenumber in the entire range of the spectrum [11,13]. The following table (Table 1) shows part of the measured values, and Figure 5 shows a graphic interpretation of the measured values with a detailed display of the highest peaks. The number of recorded values depends on the settings of the selected device, and in this case, it is 1455.

Where PVB 10 represents a material with 10 percent of textile fibre content, PVB 20 represents a material with 20 percent of textile fibre content, etc. It can be seen from the graphic display that the absorption values have the same shape of the curve. This assumption will be the starting point in the search for a mathematical model so that the qualitative indicators are sufficient for all the investigated materials.

### 2.2. Methods

From the point of view of using mathematical modelling for composite materials, procedures are traditionally used where distribution functions, such as searching for experimentally obtained data using Gaussian, exponential, Laplacian, Rayleigh, Weibull, Wigner, and Pareto functions [14]. Such an approach is advantageous because the given mathematical apparatus already exists and is built into several application programs. In the presented contribution, we will use the Gaussian regression function (GPR) for the design of the model because of its use for both regression and classification problems. It is a statistical approach that attempts to approximate the input–output mapping from the measured data using a Gaussian process model [15] and provides an explicit measure of uncertainty that helps quantify the reliability of the measurement [16,17]. These methods use non-parametric functions based on probabilistic models [18]. A key advantage is the ability to model complex functions without overfitting or underfitting. It can handle non-linear relationships between input and output variables and captures uncertainty in the forecast. Gaussian regression is also considered a powerful and adaptive machine-learning technique that combines different machine-learning tasks [19]. This enables its use for a wide range of applications. The authors [15] evaluated the suitability of the GPR technique for predicting reservoir porosity and permeability, finding that GPR models generated faster than comparable results with widely used ANN methods. This method is also used in composite materials, e.g., in [20] to predict the compressive strength of polyurethane PC.

**Figure 6:**
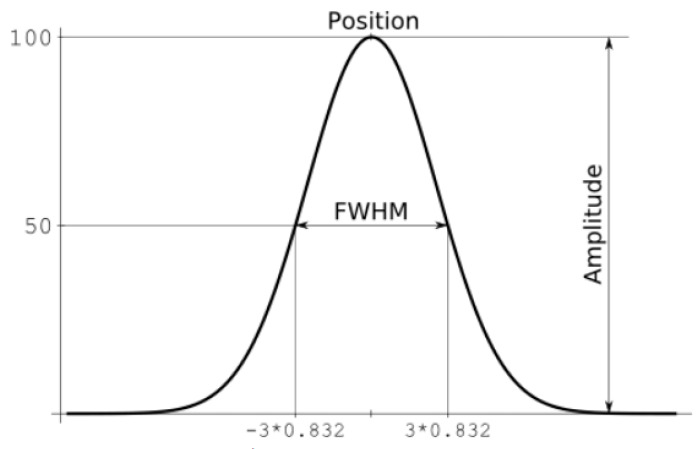
Schematic display of individual coefficients.

We will use the model interpreted by the equation:

(1) $$y=\sum_{i=1}^{n}a_{i}e^{\left[-{\left(\frac{x-b_{i}}{c_{i}}\right)}^{2}\right]}$$

where *a* is the amplitude, *b* is the centre of gravity (location), *c* is related to the peak width, and *n* is the number of peaks. A schematic representation of the individual coefficients is shown in the Figure 6, where the variable named FWHM is $6*\sqrt{log2}$ [21].

#### 2.2.1. Qualitative Assessment

The following metrics were used to assess the individual variants obtained by changing the coefficients:

- Mean Absolute Error (MAE)—The mean absolute error indicates the average difference between the actual and predicted values. The closer the MAE value is to 0, the more appropriate the prediction [22,23,24].

(2) $$MAE=\frac{1}{n}\sum_{i-1}^{n}\left|y_{i}-{\hat{y}}_{i}\right|$$

where *n* is the number of values, *y*_i_ is the measured value, and ${\hat{y}}_{i}$ as a predicted value.

- R-squared (R^2^)—The determination index is defined as the proportion of the variability that the regression model can describe to the total variability of the observed variable. Its values are from 0 to 1, a higher value of this index indicates a more suitable model [25].

(3) $$R^{2}=1-\frac{\sum_{i=0}^{n}{\left(y_{i}-{\hat{y}}_{i}\right)}^{2}}{\sum_{i=1}^{n}(y_{i}{-{\bar{y}}_{i})}^{2}}$$

where *n* is number of values, *y_i_* is the measured value, the ${\hat{y}}_{i}$ is the predicted value, and $\bar{y}$ is the arithmetic mean.

- Root Mean Square Error (RMSE)—The root mean squared error measures the magnitude of the error between two data sets [20,26]. It compares the predicted value with the observed or known value. The lower the value of the index, the more suitable the model.

(4) $$RMSE=\sqrt{\frac{1}{n}\sum_{i=1}^{n}{\left(y_{i}-{\hat{y}}_{i}\right)}^{2}}$$

where *n* is number of values, *y_i_* measured value a ${\hat{y}}_{i}$ predicted value.

#### 2.2.2. Tools

The Matlab R2023 environment was used as the basic environment for the design of the mathematical model. This application is widely used in various fields requiring numerical calculations and data analysis due to its powerful tools, which include additional tools specific to its field of use. Selected key functions include graphics, data visualization, programming, and the creation of user interfaces [27,28]. In this article, we will use the curve fitting toolbox, which allows users to search for various mathematical models in order to describe the measured data as best as possible. It provides a graphical user interface (GUI) for selecting models, defining customization options, and visualizing results. Its basic functions include a set of tools containing:

- A large library of predefined models, including linear and non-linear models, and the ability of the user to create their own models;
- Powerful data visualization tools that help users identify trends and outliers in data;
- Model selection tools that allow you to compare the performance of different models and choose the best one for the monitored data;
- Several methods for estimating model parameters, including least squares regression, non-linear regression, and maximum likelihood estimation,
- A toolbar allowing the user to export analysis results to variables of the Matlab workspace, as well as to files in various formats, including Excel, CSV, and LaTeX [29].

To search for a generalized mathematical model, we will proceed in the following way:

- We experimentally obtain absorption values for individual composite samples and data preprocessing;
- Using the curve fitting toolbox, we select the function and compare the values of the R^2^ coefficient;
- We analyse the coefficients of the selected model for the shape of the curve and qualitative indicators;
- Implementing changes in the individual coefficients of the selected model based on the analysis, we evaluate the achieved values of the qualitative coefficients of the model using the Matlab computing environment;
- We present the final mathematical model.

## 3. Model Proposal

The first step is to select the function that will form the basis for the search function, using the curve fitting tool in Matlab. The modified data were imported into the basic Matlab environment, and the relevant variables were prepared for the use of the toolbox. The x-coordinate is “Wavenumber”, and the y-coordinate is the obtained measured absorption data [29]. By successively searching for the best model, the “Gaussian” option was selected. Based on the graphical interpretation of the measured data, we recorded 5 significant peaks. This assumption was subsequently used when setting the parameters of the selected “Gaussian” type in the curve fitting toolbox environment in the Matlab application. When using the specified type, the obtained values of the coefficient of determination are listed in Table 2. This function was subsequently used for all material variants, and the value of the quality coefficient R2, RMSE, and SSE was compared. Table 2 shows the specified values.

This function was subsequently used for all material variants, and the value of the quality coefficient R^2^, RMSE, and SSE were compared. Table 2 shows the specified values.

The values in the Table 2 show that the selected mathematical model is the least suitable for the material containing 10% textile fibre and the most suitable for the material with 20% textile fibre content. This material will be chosen as the basic one. In this environment, there are displayed ranges of individual coefficients of the selected model, its equation, and the statistical characteristics interpreting the model’s suitability. Using the save to workspace option, the model is saved in the basic environment [30,31]. In order to be able to update the model in case of new data, we also save its function, which the given environment is capable of generating.

The generated equation has the form:

(5) $$y=a_{1}e^{{\left(-((x-b_{1})/c_{1}\right)}^{2}}+a_{2}e^{{\left(-((x-b_{2})/c_{2}\right)}^{2}}+a_{3}e^{{\left(-((x-b_{3})/c_{3}\right)}^{2}}+a_{4}e^{{\left(-((x-b_{4})/c_{4}\right)}^{2}}{+a}_{5}e^{{\left(-((x-b_{5})/c_{5}\right)}^{2}}$$

**Figure 7:**
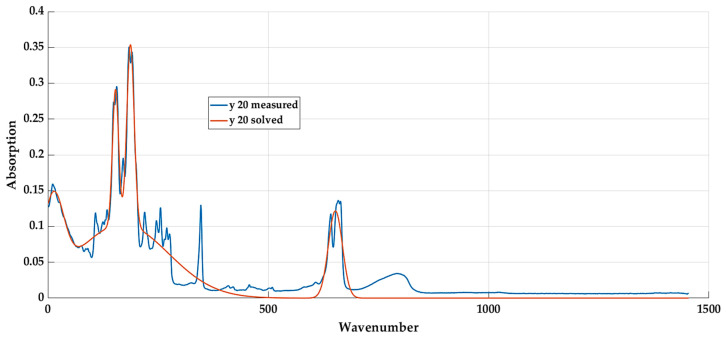
Basic model in the curve fitting toolbox.

Figure 7 interprets the graphic display of measured values and values obtained using the selected function for a material with a 20% textile fibre content.

The task of further investigation will be to determine whether it is possible to increase the value of the qualitative indicator R^2^ by changing the coefficients of the generated function.

**Figure 8:**
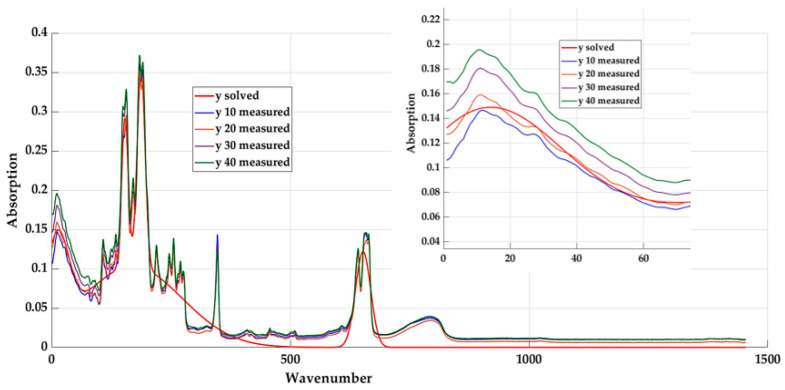
Graphic course of measured and calculated values for the selected material with detail of the introductory part.

Figure 8 represents a graphical interpretation of the calculated values based on Equation (5) for the base material (with 20% content—*y* solved) and the measured values of the other materials.

We will determine the values of the quality indicators for all these materials. The results for individual quality coefficients R^2^, MAE, and RMSE are presented in Table 3. Their calculations were made on the basis of Equations (2)–(4). In order to perform comparisons of the individual variants of the investigated models, we used the “overall average” value, which is the result of the arithmetic average of the R^2^ value and “range”.

Based on the graphic display and the values of the qualitative indicators, it is shown that the use of the given model is insufficient, especially for materials with a 40% textile fibre content. The goal is to change the individual coefficients to modify this model so that it is more suitable for all materials.

### Analysis of the Influence of Individual Coefficients

**Figure 9:**
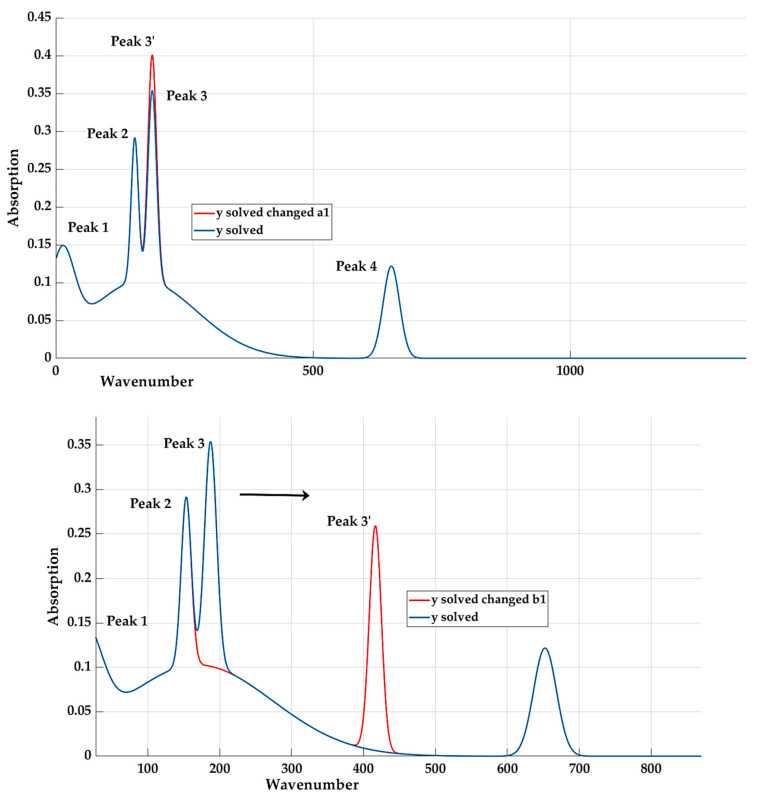

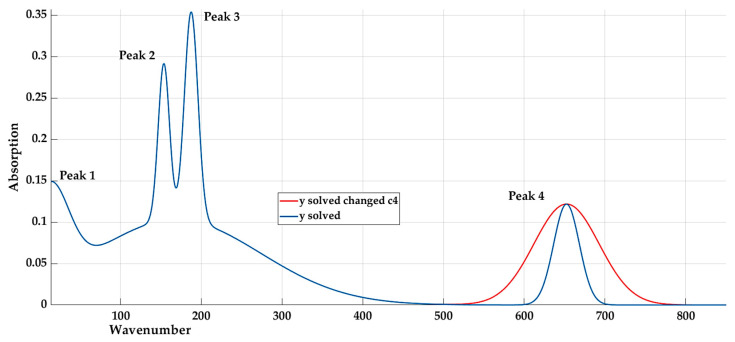
The influence of changes in selected coefficients on the shape of the curve.

In order to be able to purposefully adjust the resulting shape of the model, it is necessary to verify the influence of individual coefficients on the resulting values and the shape of the curve. Figure 9 graphically represents the effect of changing (increasing) the value of the coefficients *a*_1_, *b*_1_, and *c*_4_.

**Figure 10:**
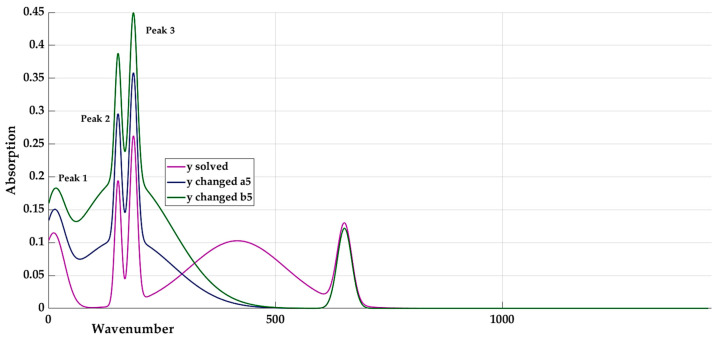
The influence of coefficients *a_5_* and *b_5_*.

The influence of coefficients *a_5_* and *b_5_* is shown in Figure 10. The coefficient *c_5_* has almost no influence on the resulting curve.

A schematic representation of the influence of individual coefficients when their value is increased is expressed in Table 4.

**Figure 11:**
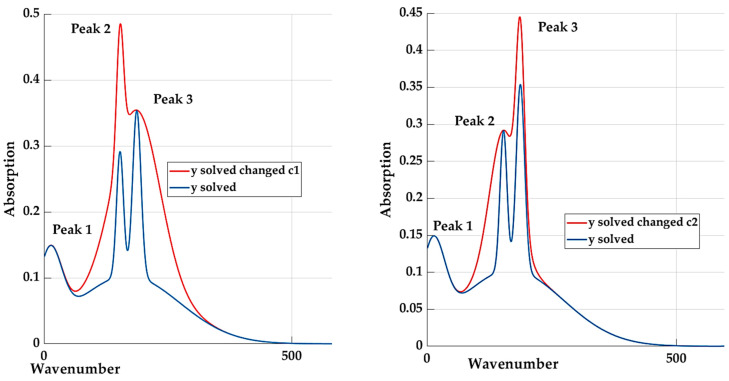
The effect of changing the coefficients *c_1_* nd *c_2_* on the shape of the curve.

A graphic representation of the influence of coefficients *c_1_* and *c_2_* is shown in Figure 11.

The analysis confirmed that coefficient *a*_1_ has an effect on peak 3 and coefficient *a_3_* has an effect on peak 1.

## 4. Results and Discussion

By changing the parameters, we will try to find a function where the values of quality indicators for all materials would be approximately at the same level. The resulting values will be compared with Table 3. From the graphic interpretation of the original results (Figure 9) and the schematic representation (Figure 6), it can be assumed that we can achieve better results by increasing the amplitude. Based on the previous analysis of the influence of individual coefficients, we will focus on the change of coefficients *a (*Table 5). When creating a model in the curve fitting toolbox [31], the minimum and maximum values of individual coefficients are available, while the value used represents their arithmetic mean.

Individual scenarios:

1. “Increasing” the values of the coefficients *a*_1_, *a*_2_, *a*_3_, and *a*_4_ (compared to the original ones), we can obtain a better adaptation of the curve to all values. We do not consider the coefficient *a*_5_ yet, since its change affects the entire curve and not just its peaks. When calculating the assumed values based on Equation (5), we use the maximum values of these coefficients. The results are shown in Table 6.

**Figure 12:**
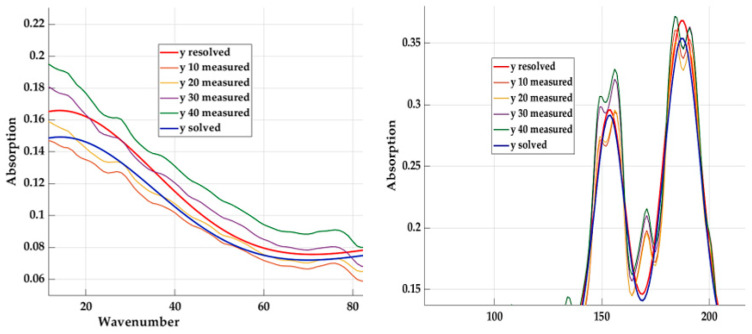
The shape of the curve after changing the selected coefficients (details of peaks).

R^2^ values decreased for materials with 10% and 20% textile fibre, and vice versa, the values for materials with 30% and 40% increased. The overall average value of R^2^ increased, indicating a better fit of the model for all curves. Figure 12 represents the details of the graphical display of the curve after changing the coefficients.

**Figure 13:**
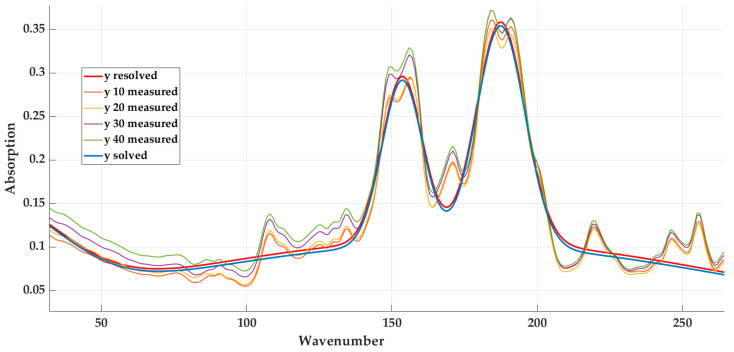
Part of the curve after changing the selected coefficient.

2. However, we can also consider the use of coefficient *a*_5_, since by increasing it, we can “increase” the entire curve (Figure 9). We set its maximum value. The numerical values of the qualitative indicators are shown in Table 7 and presented in the Figure 13.

Comparing the values of R^2^ with the original values (Table 4) shows that even with this change, the value of this coefficient will increase slightly. So, there is some improvement. However, if we were to increase this value slightly, the calculation shows that the overall average value of R^2^ [30] will be lower than in the previous case. This is due to the fact that an increase in the value of this coefficient affects not only the peak itself but the entire course of the curve, and this is the reason for the lower value [31].

3. Based on the analysis, we could assume an improvement when changing all coefficients and also setting the value of the coefficient *b_5_* to the maximum value (Table 8).

The R^2^ values again show an improvement not only compared to the original model but also compared to strategy 1. As the analysis of the influence of the coefficients showed, changing the coefficients c does not imply an improvement. Therefore, they were not considered even in individual cases.

The form of the equation with the table of the resulting values of the coefficients follows:

(6) $$y=a_{1}e^{{\left(-((x-b_{1})/c_{1}\right)}^{2}}+a_{2}e^{{\left(-((x-b_{2})/c_{2}\right)}^{2}}+a_{3}e^{{\left(-((x-b_{3})/c_{3}\right)}^{2}}+a_{4}e^{{\left(-((x-b_{4})/c_{4}\right)}^{2}}{+a}_{5}e^{{\left(-((x-b_{5})/c_{5}\right)}^{2}}$$

**Figure 14:**
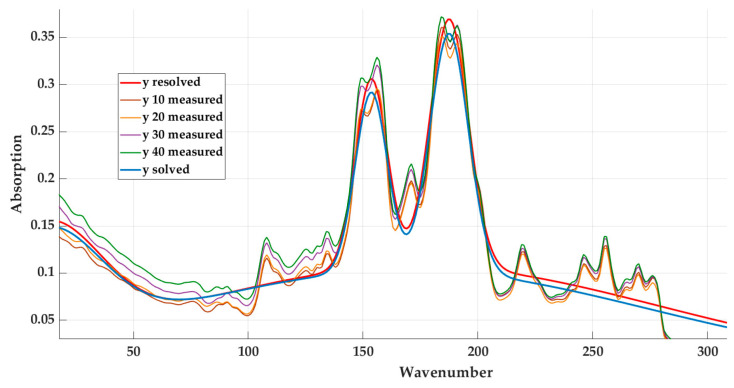
Display of modified model (*y resolved*) and original model (*y solved*).

The following Figure 14 shows the original model (*y solved*) and the model obtained by modifying the coefficients (*y resolved*).

Based on Table 9, scenario No. 3 and the coefficients listed in Table 10 were selected for the final equation. Figure 14 shows the course of the originally determined curve and the curve calculated using the determined coefficients.

As part of the search for an optimal variant for determining the final mathematical model, options were also evaluated for the case where the basis would be a model generated for a material with a 40% textile fibre content. The value of the coefficient of determination R^2^ was the second highest. It was necessary to “reduce” the curve compared to the resulting model, but even when changing the value of the coefficients affecting the curve, better results were not achieved than were presented.

## 5. Conclusions

Composite materials are gaining more and more applications in various fields. The effort to recycle used materials and combine them with other materials creates unique properties. However, the application of such materials strongly depends on their properties. The analysis of these properties becomes an integral part in the field of their use. This paper investigated four samples of composite materials with different content of textile fabrics. The goal was to specify one mathematical model that would together determine the absorption value and the possible impact on possible corrosion for these materials with sufficient accuracy. The following results were achieved through the analysis of experimental data and the chosen methods:

- The GPR technique was chosen, which has the advantages of easy implementation and flexibility compared to other techniques from the field of neural networks and supporting computing environments;
- Based on the analysis of the influence of the individual coefficients on the behaviour of the curve of the mathematical model, an equation was determined with the specified values of the individual coefficients.
- Individual scenarios were compared, where the comparison parameter was the value of the coefficient of determination and the range.
- Scenario number 3 turned out to be the most suitable, where, for individual materials using the given equation, the value of the coefficient of determination for 10% is 0.90657, for 20% is 0.925851, for 30% is 0.91472, and for 40% is 0.90781. We can consider these values to be sufficient. The given variant also supports the range value that has the lowest value, meaning the smallest difference between the largest and smallest value.
- The final equation was specified, which predicts the absorption values for the given material with sufficient accuracy (around 90%), while the percentage content of the textile fibre is not decisive.

Absorption is an important property to consider in the design and manufacture of composite materials with textile fibres. By controlling the absorption properties of the material, it is possible to improve its performance and durability in a wide range of applications. For the future work, we would like to observe various techniques to minimize the effects of absorption in composite materials. These techniques can help reduce the absorption of moisture and other fluids and improve the durability and performance of the composite material.

## Figures and Tables

**Table 1:** Partial list of values.

yi_10	yi_20	yi_30	yi_40
0.106227	0.127227	0.146199	0.169929
0.107909	0.127779	0.147441	0.169904
0.112041	0.129668	0.149271	0.168662
0.117294	0.132858	0.153041	0.1712
0.120815	0.136888	0.157586	0.174762
0.123528	0.141113	0.16032	0.17854
0.129636	0.144677	0.16399	0.184941
0.135411	0.14888	0.17018	0.189669
0.138901	0.153937	0.175523	0.192442

**Table 2:** Values of observed coefficients.

Fibre Content	10	20	30	40
R^2^	0.9107	0.9291	0.9181	0.9207
RMSE	0.0168	0.0152	0.0173	0.0175
SSE	0.4073	0.3309	0.4310	0.4417

**Table 3:** The results of selected coefficients.

Fibre Content	10	20	30	40	Overall Average	Range
R^2^	0.90790	0.92912	0.90855	0.89891	0.91112	0.03021
MAE	0.00909	0.00670	0.01201	0.01385		
RMSE	0.01699	0.01508	0.01819	0.01968		

**Table 4:** The effect of changes in individual coefficients.

Order	Peak	*a*	*b*	*c*
1	3	↑	→	Expands the area of peak 2 and 3, while increasing peak 2
2	2	↑	→	Expands the area of peak 2 and 3, while increasing peak 3
3	1	↑	→	Will widen and slightly raise peak 1
4	4	↑	→	Expand peak 4
5	curve	Will increase the peaks 1, 2, 3 and expands the starting parts of the graph	Will reduce the peaks 1, 2, 3 and increases the ramp between the peaks 3 and 4	Will slightly increase peak 1 and the ramp between 2 and 4

**Table 5:** Generated min and max values for coefficient *a*.

Coefficient	*a*_1_		*a*_2_		*a*_3_		*a*_4_		*a*_5_	
	min	max	min	max	min	max	min	max	min	max
	0.2429	0.2633	0.1791	0.201	0.1068	0.1227	0.115	0.1287	0.09846	0.1072

**Table 6:** Replacing the value of the coefficients “a” with their maximum value.

Fibre Content	10	20	30	40	Overall Average	Range
R^2^	0.90651	0.92789	0.91304	0.90535	0.91319	0.02254
MAE	0.00840	0.00601	0.01133	0.01316		
RMSE	0.01712	0.01523	0.01774	0.01904		

**Table 7:** Setting the value of coefficient *a_5_* to max.

Fibre Content	10	20	30	40	Overall Average	Range
R^2^	0.90034	0.92095	0.91496	0.91167	0.91198	0.02061
MAE	0.00662	0.00423	0.00955	0.01139		
RMSE	0.01768	0.01593	0.01754	0.01839		

**Table 8:** Setting the value of coefficients, *a* (all) to max. and *b_5_* max.

Fibre Content	10	20	30	40	Overall Average	Range
R^2^	0.90657	0.92585	0.91472	0.90781	0.91374	0.01927
MAE	0.00755	0.00515	0.01047	0.01231		
RMSE	0.01712	0.01543	0.01757	0.01879		

**Table 9:** Summary of results.

Scenario	Overal Average	Range	RMSE	MAE
1	0.91320	0.02253	0.01728	0.00973
2	0.91198	0.02060	0.01739	0.00795
3	0.91374	0.01927	0.01723	0.00887

**Table 10:** Coefficients values.

Coefficients					
*a*_1_	0.2633	*b*_1_	1116.4731	*c*_1_	45.5136
*a*_2_	0.201	*b*_2_	985.71317	*c*_2_	38.0459
*a*_3_	0.1227	*b*_3_	438.99169	*c*_3_	129.489
*a*_4_	0.1287	*b*_4_	2909.8266	*c*_4_	86.7078
*a*_5_	0.1072	*b*_5_	1064	*c*_5_	573.9658

## Data Availability

Not applicable.
